# A Comparison of the Long-Term Effects of Lanthanum Carbonate and Calcium Carbonate on the Course of Chronic Renal Failure in Rats with Adriamycin-Induced Nephropathy

**DOI:** 10.1371/journal.pone.0097859

**Published:** 2014-05-20

**Authors:** Tsuyoshi Takashima, Toru Sanai, Motoaki Miyazono, Makoto Fukuda, Tomoya Kishi, Yasunori Nonaka, Mai Yoshizaki, Sae Sato, Yuji Ikeda

**Affiliations:** 1 Department of Nephrology, Faculty of Medicine, Saga University, Saga, Japan; 2 Department of Nephrology, Fukumitsu Hospital, Fukuoka, Japan; 3 Department of Nephrology, Ureshino Medical Center, Ureshino, Japan; Instituto Nacional de Cardiologia I. Ch., Mexico

## Abstract

Lanthanum carbonate (LA) is an effective phosphate binder. Previous study showed the phosphate-binding potency of LA was twice that of calcium carbonate (CA). No study in which LA and CA were given at an equivalent phosphate-binding potency to rats or humans with chronic renal failure for a long period has been reported to date. The objective of this study was to compare the phosphate level in serum and urine and suppression of renal deterioration during long-term LA and CA treatment when they were given at an equivalent phosphate-binding potency in rats with adriamycin (ADR)-induced nephropathy. Rats were divided into three groups: an untreated group (ADR group), a CA-treated (ADR-CA) group and a LA-treated (ADR-LA) group. The daily oral dose of LA was 1.0 g/kg/day and CA was 2.0 g/kg/day for 24 weeks. The serum phosphate was lower in the ADR-CA or ADR-LA group than in the ADR group and significantly lower in the ADR-CA group than in the ADR group at each point, but there were no significant differences between the ADR and ADR-LA groups. The serum phosphate was also lower in the ADR-CA group than in the ADR-LA group, and there was significant difference at week 8. The urinary phosphate was significantly lower in the ADR-CA group than in the ADR or ADR-LA group at each point. The urinary phosphate was also lower in the ADR-LA group than in the ADR group at each point, and significant difference at week 8. There were no significant differences in the serum creatinine or blood urea nitrogen among the three groups. In conclusion, this study indicated the phosphate-binding potency of LA isn’t twice as strong as CA, and neither LA nor CA suppressed the progression of chronic renal failure in the serum creatinine and blood urea nitrogen, compared to the untreated group.

## Introduction

Effective control of the phosphate overload in patients with chronic kidney disease (CKD) is now recognized as an important target for reducing the high mortality rate associated with this condition [1], [2], and current practice guidelines recommend aggressive treatment of hyperphosphatemia to achieve lower serum phosphorus targets.

A low-phosphate diet has been shown to prevent the progression of experimental renal disease [3]–[5]. However, the administration of a low-phosphate diet is consistently associated with a decreased intake of protein, calories and other nutrients, the absence of which results in nutritional deficits, especially in cases of progressive renal disease with massive proteinuria.

Phosphate binders are administered in order to decrease the serum phosphate levels by adsorbing phosphate in the intestine. They bind phosphate, form insoluble products, and selectively suppress the intestinal absorption of phosphate without leading to a deficiency of other nutritional elements. Therefore, oral administration of the phosphate-binders may be utilized to prevent renal deterioration, without causing subsequent malnutrition. Lumlertgul et al. reported that a phosphate binder (dihydroxyaluminum aminoacetate) suppressed the progression of chronic renal failure (CRF) in rats with 5/6 nephrectomy [6]. Sanai et al. reported that calcium carbonate (CA) and aluminum hydroxide suppressed the progression of CRF in rats with adriamycin (ADR)-induced nephropathy [7]. ADR-induced nephropathy in rats is a commonly used model of chronic renal disease, characterized by persistent proteinuria and progressive reduction in renal function leading to terminal renal failure [8]. Morphological lesions resemble focal glomerular sclerosis. Epithelial degeneration develops as an initial lesion in the kidney, and progresses to irreversible glomerular sclerosis and tubulointerstitial changes [9].

Lanthanum carbonate (LA) is a calcium-free oral phosphate binder that can control hyperphosphatemia without adding to the patient’s calcium load [10]. Previous studies have shown a high phosphate binding capacity of LA [11]. According to the study by Hutchison et al. of 800 hemodialysis patients who were randomized to receive either LA or CA, where the dose was titrated to achieve control of the serum phosphate level [12], the phosphate-binding potency of LA was twice as strong as that of CA at the same weight [13]. Hutchison et al. also reported that at a dose of 1.0 g/kg/day of LA rapidly reduced the mean urinary phosphate excretion and sustained the reduction to the end of the six-week treatment period much better than a dose of 1.0 g/kg/day of CA in rats with 5/6 nephrectomy [14].

To the best of our knowledge, no study in which LA and CA were given at an equivalent phosphate-binding potency in rats or humans with CRF for a long period has been reported to date. The objective of this study was to compare the long-term effects of equivalent doses (in terms of potency) of LA and CA in terms of controlling the serum phosphate level and suppressing the renal deterioration in rats with ADR-induced nephropathy.

## Materials and Methods

### Ethics Statement

All experiments were approved by the Animal Experimental Ethical Committee of Saga University (Permit Number: 24-042-0), and were conducted in compliance with our institutional guidelines and with international standards for the manipulation and care of laboratory animals. All of the surgeries were performed under diethyl ether anesthesia, and all efforts were made to minimize suffering.

### Animal Model

**Figure 1:**
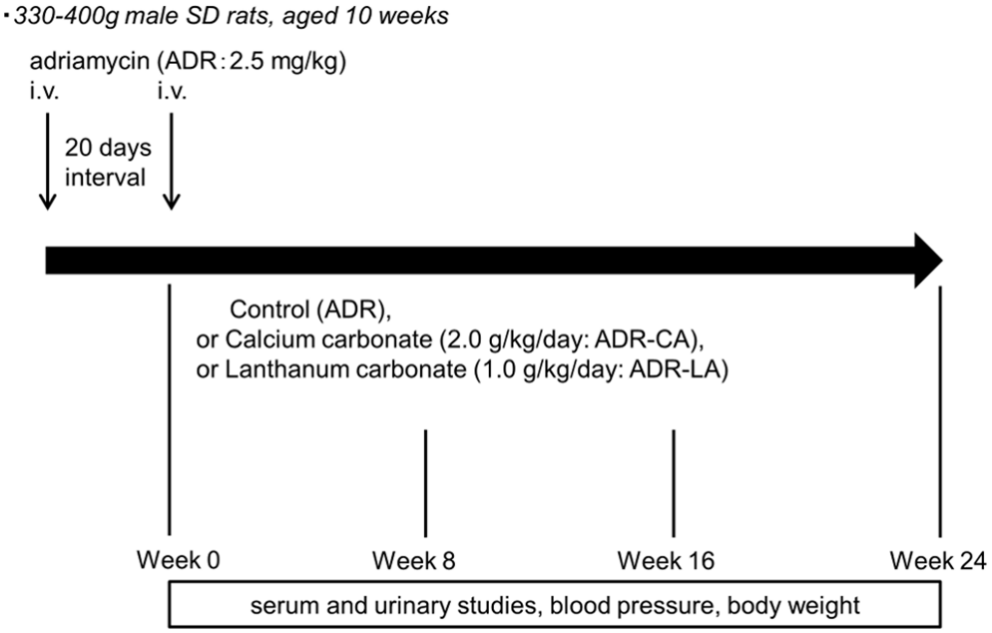
An outline of the experimental protocol. i.v.: intravenous injection.

ADR (2.5 mg/kg) was intravenously administered to male Sprague-Dawley rats (Charles River Breeding, Kanagawa, Japan). The rats, aged 10 weeks and weighing 330–400 g, were treated with ADR twice at a 20-day interval according to the method reported previously [8] (Figure 1). The rats were then randomly divided into three groups: a group without a phosphate binder (ADR group, n = 18), an ADR-CA-treated group (ADR-CA group, n = 18) and an ADR-LA-treated group (ADR-LA group, n = 18). LA (FOSRENOL, Bayer Yakuhin, Ltd., Osaka, Japan) or CA (precipitated calcium carbonate, Asahi Kasei Pharma Corp., Tokyo, Japan) was mixed with standard chow (MF, Oriental Yeast Co., Ltd., Tokyo, Japan), containing 23.1% protein, 0.19% sodium, 1.07% calcium and 0.83% phosphorus, and 359 Cal/100 g. There were no significant differences among the groups. The phosphate binder treatments were started after the second administration of ADR (week 0). The daily dose of LA was 1.0 g/kg/day and that of CA was 2.0 g/kg/day throughout the experiment. The diet was adjusted to match that of the group with minimal ingestion, as determined at weekly intervals according to the consumption, in order to maintain the same calorie and protein intake in all groups. All groups had free access to tap water. The body weight, blood pressure, blood chemistry parameters and a 24-hour urinary collection were examined every eight weeks until week 24 after the second administration of ADR. The serum fibroblast growth factor (FGF)-23 levels and the serum parathyroid hormone (PTH) levels were determined at weeks 0, 8 and 24. The serum 1,25-(OH)_2_ vitamin D levels were determined at weeks 0 and 24. All surviving rats were sacrificed at week 24 and expression levels of Klotho in renal tissue were analyzed by real-time PCR.

### Biochemical Analysis

The urinary protein from a 24-hour collection was measured using the pyrogallol red method. The urinary phosphate from the 24-hour collection was measured using the molybdate direct method, and the urinary calcium from a 24-hour collection was measured using the arsenazo III method. The systolic blood pressure was measured in the conscious state by the tail cuff method. Blood samples were drawn from a tail vein for determinations of the serum creatinine, phosphate, blood urea nitrogen (BUN) and total cholesterol level by enzymatic methods, the calcium level by use of orthocresolphthalein complex one, the total protein by the Biuret reaction, and albumin was detected by bromcresol green staining. The level of FGF-23 was determined by an ELISA kit (Kainos Laboratories, Inc., Tokyo, Japan). The level of serum PTH was measured with the rat PTH-EIA kit (Peninsula Laboratories, LLC., San Carlos, CA, USA). A radioimmunoassay kit (TFB, Inc., Tokyo, Japan) was used for the measurement of the serum 1,25-(OH)_2_ vitamin D.

### Kidney

Kidneys were harvested and total RNA was isolated as earlier by RNeasy Protect Mini kit (QIAGEN K. K., Tokyo, Japan) using the manufacturer’s protocol. Expression levels of Klotho were analyzed by real-time PCR using the manufacturer’s protocol (Applied BioSystems, Inc.) and commercially available primers from Applied BioSystems, Inc. Glyceraldehyde 3-phosphate dehydrogenase (GAPDH) served as an internal control, and all results are expressed as the ratio of Klotho RNA/GAPDH. TaqMan Gene Expression Master Mix (Applied Biosystems, Inc., Carlsbad, CA, USA) was used to perform quantitative PCR. PCR conditions for all experiments were 2 minutes at 50°C, 10 minutes at 95°C, followed by 40 cycles of 15 seconds at 95°C and 1 minute at 60°C. The data were collected and analyzed by the Step One Plus Real Time PCR System and software (Applied BioSystems). All primer sets were tested for specific amplification of mRNA by parallel analyses of controls that included omitting RT and resulted in no fluorescent signal detection. The 2^−ΔΔCt^ method described by Livak was used to analyze the data.

### Statistical Analysis

Differences between multiple time points for each study group were determined by the Friedman test, followed by a Wilcoxon signed-rank test with Bonferroni correction. Comparisons between the study groups for each time point were assessed using a Kruskal-Wallis test, followed by a Mann-Witney *U*-test in combination with Bonferroni correction. A two-sided P-value was considered to be significant for values <0.05. The data are expressed as the means ± SD. All analyses were done using the SPSS 22.0 (IBM Japan, Tokyo, Japan).

## Results

One of the eighteen rats died between weeks 8 and 16, and five rats died between weeks 16 and 24 in the ADR group. Five of the eighteen rats in the ADR-CA group died between weeks 16 and 24. Three of the eighteen rats died between weeks 8 and 16, and four rats died between weeks 16 and 24 in the ADR-LA group. All of the rats were considered to have died of uremia, because the daily food intake and body weight gradually decreased, and the serum creatinine level increased.

**Figure 2:**
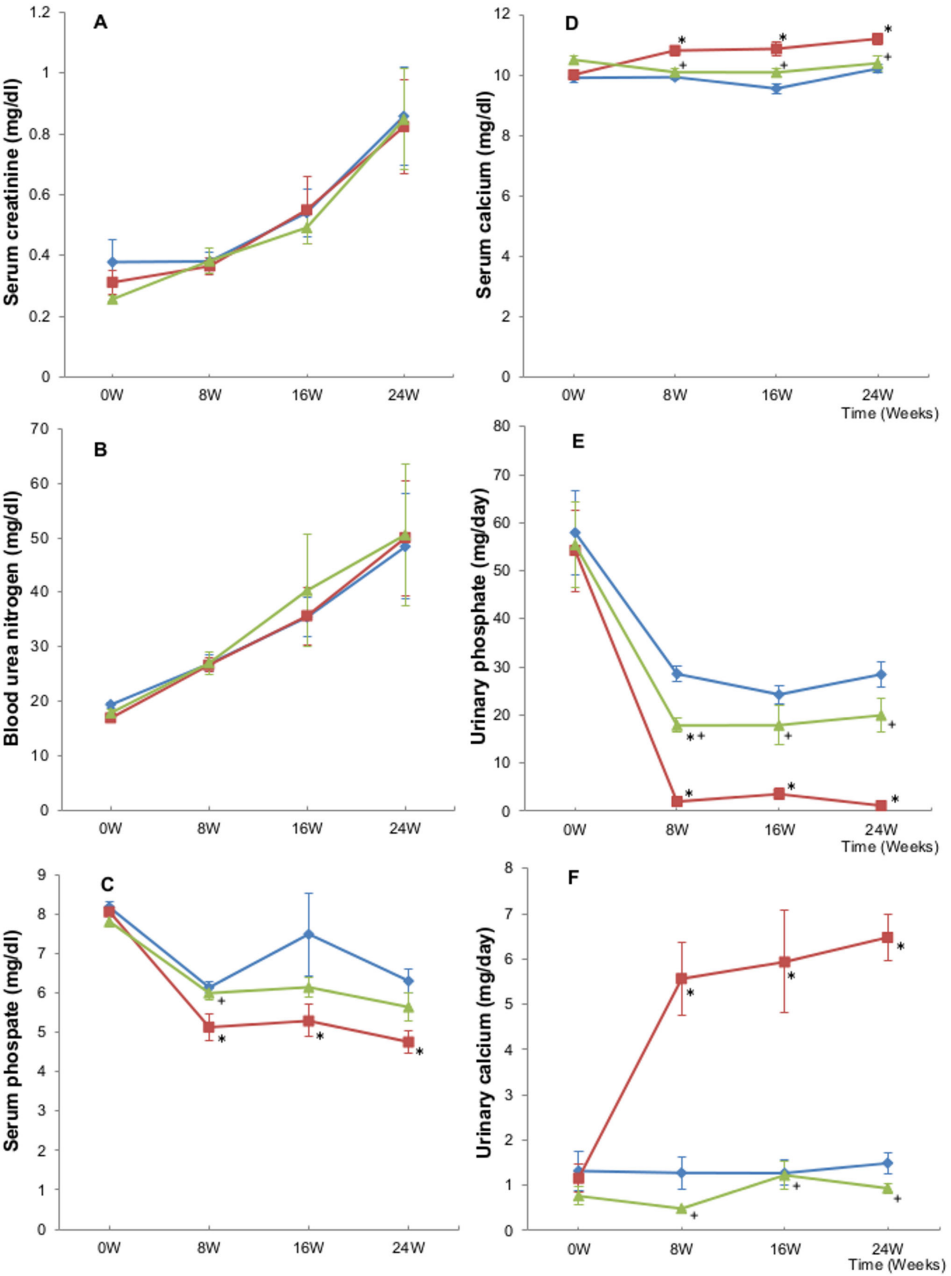
The serum and urine data. A: serum creatinine, B: blood urea nitrogen, C: serum phosphate, D: serum calcium, E: urinary phosphate, F: urinary calcium. The data are expressed as the means ± SEM. ♦ = ADR group, ▪ = ADR-CA group, ▴ = ADR-LA group. *p<0.05 or less, vs. ADR group at the same time point, ^+^p<0.05 or less vs. ADR-CA group at the same time point.

The serum creatinine and BUN levels increased progressively, but there were no significant differences among the three groups at any of the time points (Figures 2A, 2B).

The serum phosphate level was lower in the ADR-CA group and in the ADR-LA group compared to the ADR group at each point (Figure 2C). The serum phosphate level was significantly lower in the ADR-CA group than that in the ADR group at each point (p<0.05). However, while there were some differences in the serum phosphate level at some points in the ADR and ADR-LA groups, none of these differences was significant. The serum phosphate level was lower in the ADR-CA group compared to the ADR-LA group at each point, and there was significant difference at week 8 (p<0.05).

The serum calcium level in the ADR-CA group was significantly higher than that in the ADR group (p<0.01) or in the ADR-LA group (p<0.05) at each point (Figure 2D). In addition, there were no significant differences in the serum calcium level at any point between the ADR and ADR-LA groups.

The urinary phosphate excretion decreased to a negligible level in the ADR-CA group (Figure 2E). The urinary phosphate level was significantly lower in the ADR-CA group than that in the ADR group or in the ADR-LA group at each point (p<0.01). The urinary phosphate level was lower in the ADR-LA group than in the ADR group at each point, and there was significant difference at week 8 (p<0.01).

The urinary calcium level was significantly higher in the ADR-CA group than that in the ADR group or in the ADR-LA group at each point (p<0.01) (Figure 2F). There were no significant differences in the urinary calcium level at any point between the ADR and ADR-LA groups.

The other data are shown in Table 1. The body weight gradually increased in all of the groups, but there were no significant differences in body weight among the three groups at each point. A mild to moderate elevation in the systolic blood pressure was observed in all three groups, but there were no significant differences in the systolic blood pressure among the three groups at each point. In terms of the serum biochemical data (total protein, albumin, total cholesterol, PTH, and 1,25-(OH)_2_ vitamin D), there were no significant differences among the three groups at each point. The serum FGF-23 level was lower in the ADR-CA group at weeks 8 and 24 than that in the ADR group or in the ADR-LA group, and was significantly lower in the ADR-CA group at week 8 than that in the ADR group (p<0.05). The proteinuria increased in all three groups, but there were no significant differences among the three groups at any of the time points.

**Table 1:** The body weight, blood pressure, and biochemical data in the different groups.

Index	Group	Week 0	Week 8	Week 16	Week 24
**Body weight (g)**	**ADR**	445.3±40.9	520.3±64.0^a^	521.7±91.3^a^	578.0±81.8^a^ ^, c^
	**ADR-CA**	437.1±37.5	485.4±55.0^a^	491.1±66.8^a^ ^, b^	508.9±58.2^a^ ^, c^
	**ADR-LA**	440.2±28.4	495.9±48.0^a^	510.7±57.6^a^ ^, b^	537.3±49.3^a^
**Systolic blood pressure (mmHg)**	**ADR**	119.0±16.5	140.4±17.6	147.3±12.8^a^	140.3±18.5
	**ADR-CA**	124.0±15.5	139.2±16.9	144.1±20.2	153.5±16.4^a^
	**ADR-LA**	121.2±19.8	142.5±18.3	137.8±18.5	146.1±21.0
**Serum creatinine (mg/dL)**	**ADR**	0.38±0.36	0.38±0.14^a^	0.54±0.31^a^	0.86±0.56^a^ ^, b, c^
	**ADR-CA**	0.31±0.19	0.37±0.14	0.55±0.44	0.82±0.54^a^ ^, b, c^
	**ADR-LA**	0.26±0.04	0.38±0.20	0.49±0.20^a^	0.85±0.50^a^ ^, b^
**Blood urea nitrogen (mg/dL)**	**ADR**	19.4±2.6	26.9±7.6	35.4±13.8^a^ ^, b^	48.4±33.4^a^ ^, b^
	**ADR-CA**	16.9±2.1	26.6±6.1^a^	35.7±21.7^a^	50.0±38.1^a^ ^, c^
	**ADR-LA**	17.8±0.5	27.0±10.2	40.3±39.8^a^	50.5±43.0^a^ ^, b^
**Serum phosphate (mg/dL)**	**ADR**	8.17±0.74	6.13±0.64^a^	7.49±4.20^a^	6.30±1.04^a^
	**ADR-CA**	8.06±0.52	5.13±1.62* ^,^ a	5.29±1.70* ^,^ a	4.75±1.00* ^,^ a
	**ADR-LA**	7.81±0.65	6.00±0.79+ ^,^ a	6.14±0.99^a^	5.64±1.16^a^
**Serum calcium (mg/dL)**	**ADR**	9.90±0.64	9.93±0.32	9.55±0.67	10.21±0.47
	**ADR-CA**	10.0±0.68	10.80±0.77*	10.86±0.93*	11.19±0.73*
	**ADR-LA**	10.50±0.68	10.09±0.52+	10.08±0.59+	10.38±0.89+
**Serum total protein (g/dL)**	**ADR**	6.66±0.59	8.37±2.67	7.23±1.15^a^	6.81±0.43
	**ADR-CA**	6.83±0.62	8.10±1.70	8.01±2.00	6.94±0.51
	**ADR-LA**	6.65±0.46	7.81±1.51	7.97±1.98	7.21±2.52
**Serum albumin (g/dL)**	**ADR**	4.35±0.24	2.92±0.35^a^	2.95±0.32^a^	2.95±0.23^a^
	**ADR-CA**	4.34±0.30	2.90±0.49^a^	2.82±0.35^a^	2.99±0.53^a^
	**ADR-LA**	4.42±0.23	2.85±0.31^a^	2.89±0.35^a^	2.95±0.37^a^
**Serum total cholesterol (mg/dL)**	**ADR**	69.8±10.0	402.1±184.1^a^	373.6±169.5^a^	357.0±143.1^a^
	**ADR-CA**	71.3±11.1	422.1±216.1^a^	454.5±173.0^a^	377.2±88.1^a^
	**ADR-LA**	71.1±11.0	442.1±208.6^a^	457.9±240.8^a^	348.4±171.7^a^
**Serum FGF-23 (pg/mL)**	**ADR**	403.2±107.2	620.5±117.9^a^	-	777.6±69.0^a^ ^, b^
	**ADR-CA**	416.4±90.4	444.2±123.5*	-	629.2±293.8^a^ ^, b^
	**ADR-LA**	371.9±111.6	544.0±190.5	-	780.0±128.4^a^ ^, b^
**Serum PTH (pg/mL)**	**ADR**	20.5±0.6	56.8±80.1	-	92.2±42.5
	**ADR-CA**	49.5±58.3	75.3±37.9	-	117.2±102.2
	**ADR-LA**	20.5±0.6	68.8±58.1	-	102.3±106.7
**Serum 1,25-(OH)2 vitamin D (pg/mL)**	**ADR**	275.9±119.3	-	-	81.9±55.3^a^
	**ADR-CA**	211.7±72.9	-	-	67.5±13.0^a^
	**ADR-LA**	334.1±132.5	-	-	103.1±52.3^a^
**Urinary phosphate (mg/day)**	**ADR**	57.9±42.9	28.5±4.4	24.2±7.5	28.4±9.4
	**ADR-CA**	54.2±41.5	1.97±2.69* ^,^ a	3.54±4.93* ^,^ a	1.12±1.78* ^,^ a
	**ADR-LA**	55.4±43.3	17.8±7.2* ^,^ +	17.9±16.2+	19.9±11.7+
**Urinary calcium (mg/day)**	**ADR**	1.32±1.46	1.27±1.22	1.27±0.96	1.49±0.82* ^,^ a
	**ADR-CA**	1.16±1.11	5.56±2.89* ^,^ a	5.93±4.07* ^,^ a	6.47±1.89* ^,^ a
	**ADR-LA**	0.77±0.68	0.49±0.11+	1.22±1.06+	0.94±0.37+ ^, b^
**Urinary protein (mg/day)**	**ADR**	52.1±75.3	491.4±163.1^a^	443.1±241.2^a^	504.5±111.3^a^
	**ADR-CA**	58.2±82.9	400.4±146.5^a^	404.4±123.5^a^	439.6±122.9^a^
	**ADR-LA**	49.6±62.4	459.3±191.9^a^	515.7±191.7^a^	456.1±118.3^a^

The data are expressed as the means ± SD.

*p<0.05 vs. ADR group at the same time point,

+ p<0.05 vs. ADR-CA group at the same time point.

a p<0.05 vs. Week 0 values of the same group, ^b^p<0.05 vs. Week 8 values of the same group, ^c^p<0.05 vs. Week 16 values of the same group.

Abbreviations: FGF-23, fibroblast growth factor-23; PTH, parathyroid hormone.

Expression level of Klotho in renal tissue was higher in the ADR-LA group than that in the ADR or in the ADR-CA group at week 24, and was 1.26±0.81 in the ADR group, 1.37±0.64 in the ADR-CA group and 2.48±0.88 in the ADR-LA group (Table 2). However, there were no significant differences in expression level of Klotho among the three groups (p = 0.059).

**Table 2:** Klotho expression in the kidney at week 24.

	Group	Week 24
**Klotho (RNA/GAPDH)**	**ADR**	1.26±0.81
	**ADR-CA**	1.37±0.64
	**ADR-LA**	2.48±0.88

The data are expressed as the means ± SD.

There were no significant differences among the three groups.

Abbreviations: GAPDH, Glyceraldehyde 3-phosphate dehydrogenase.

## Discussion

This animal study directly compared the long-term effects of LA and CA at an equivalent phosphate binding potency for controlling the serum phosphate level and suppressing the renal deterioration in rats with ADR-induced nephropathy.

Patients who are pre-dialysis without end-stage renal disease maintain their urine output and the ability to excrete a proportion of any phosphate that is absorbed. In the “steady-state” condition, the amount excreted in the urine is proportional to the amount absorbed. As a result, the measurement of the urinary phosphate excretion can be used as a marker of intestinal phosphate absorption, and thus, as an indicator of the phosphate binding efficacy, even when there is no change in the serum phosphate level [15]–[19].

The results of the serum and urinary phosphate in this study suggest that the phosphate binding potency of LA is not twice as strong as that CA at the same weight. As mentioned above, Hutchison et al. compared LA and CA in 800 hemodialysis patients [12], and estimated that the phosphate-binding potency of LA is twice as strong as CA at the same weight [13]. However, D’Haese et al. compared LA and CA in 98 hemodialysis patients [20], and estimated that the phosphate-binding potency of LA was 1.6 times as strong as CA at the same weight [13]. The present study supports the latter of a lower potency for LA. There may also be differences in potency between humans and rats.

This study did not indicate that either of the phosphate-binders, LA and CA, could suppress the progression of CRF, based on the serum creatinine, BUN levels, and the other parameters compared to the untreated group. As mentioned above, Sanai et al. reported in a study that used a similar protocol to the present study that CA suppressed the progression of CRF in rats with ADR-induced nephropathy [7], but the daily dose of CA was 6.0 g/kg/day throughout their experiment, and they first noted a significant difference in serum creatinine compared to the ADR group at week 34, which was longer than the duration of the present study. We believe that if we administered a higher concentration of CA or LA to rats for a longer period, they might suppress the progression of CRF, as detected by changes in the serum creatinine, BUN levels, and the other parameters compared to the ADR group.

A possible mechanism for the decrease in renal deterioration resulting from phosphate restriction may be either the prevention of metastatic calcification or a decreased energy requirement in renal tissue. Nephrocalcinosis with chronic interstitial inflammation is known to accelerate the deterioration of renal function and the progression of renal disease [21], [22]. The renal calcium content increases concomitantly with decreased renal function, a fact which has been demonstrated in biopsy materials from a wide variety of renal diseases [23]. Elevated serum phosphate seems to be the most important factor for initiating the sequence necessary for the development of nephrocalcinosis. Under these conditions, preventing phosphate retention can ameliorate the metastatic calcification through the suppression of secondary hyperparathyroidism and reduction in the calcium-phosphate product [3], [4]. The energy requirement increases in the remnant nephrons in rats with renal mass reduction, a fact which was considered to be a cause of renal injury [24]. Israel et al. also suggested that the elevation of energy metabolism was closely related to the destruction of damaged cells [25]. Prolonged phosphate depletion is known to change the renal energy metabolism. By reducing the energy requirement, the phosphate binder might exert beneficial effects on the damaged epithelial cells.

LA and CA are both commonly used to reduce the serum phosphate level during the treatment of CRF. However, CA is associated with a risk of hypercalcemia. In this study, the serum calcium level in the ADR-CA group was significantly higher than that in the ADR group or in the ADR-LA group at each point. Hypercalcemia initially leads to impaired urinary concentration [26], then to reduced renal plasma flow and glomerular filtration rates, and finally results in multiple functional and structural derangements of the kidneys. Calcium deposition in renal tissue is also a factor stimulating renal damage [27]. In addition, mineral and bone disorders in CKD patients, along with the use of calcium-based phosphate binders, may result in vascular calcification and an associated increase in mortality due to cardiovascular diseases. On the other hand, LA is associated with reduced hypercalcemic adverse events compared to calcium-based binders, although no superior effects with regard to the cardiovascular diseases have been reported so far that can justify further widespread utilization of this agent over CA [28]. In this study, expression level of Klotho in renal tissue was higher in the ADR-LA group than that in the ADR or in the ADR-CA group at week 24, but there were no significant differences among the three groups. We think there are possibilities that if we administered a higher concentration of LA (at equivalent phosphate-binding doses of LA and CA) to rats, there might be significant differences between the ADR-LA group and the other groups in expression level of Klotho, and keeping the high levels of Klotho expression in the ADR-LA group compared to the other groups suggests that LA without adding to the calcium load has a good influence on decreasing mortality.

In conclusion, this study indicated that the phosphate binding potency of LA is less than twice as strong as CA at the same weight, and neither LA nor CA suppressed the progression of CRF, as determined by the serum creatinine and BUN levels, compared to untreated rats with ADR-induced nephropathy. However, LC is a very effective phosphate binder, and previous studies have shown a very high phosphate-binding capacity (>97%) [11], [13] (Table 3), as well as low gastrointestinal absorption of LA, without serious toxic side effects [29]–[33]. Clinicians should consider the dose-response relationship for the different phosphate binders when reviewing the doses or choices of binders. A direct comparison of the binding capacities of the currently available phosphate-binders would be useful to guide clinical practice.

**Table 3:** The relative phosphate-binding coefficients for various phosphate binders.

Phosphate binder	Relative phosphate-binding coefficient per gram of compound
**Calcium carbonate (index value)**	1.0
**Calcium acetate**	1.0
**Magnesium carbonate (anhydrous weight)**	1.7
**“Heavy” magnesium carbonate (hydrated weight)**	1.3
**Aluminum hydroxide**	1.5
**Aluminum carbonate**	1.9
**Sevelamer (carbonate or hydrochloride)**	0.75
**Lanthanum carbonate**	2.0

Reproduced from Daugirdas JT et al. [13].
